# Bile duct obstruction associated with pancreatitis in 46 dogs

**DOI:** 10.1111/jvim.15879

**Published:** 2020-08-27

**Authors:** Ashley R. Wilkinson, Stefanie M. DeMonaco, David L. Panciera, Cristiane C. Otoni, Michael S. Leib, Martha M. Larson

**Affiliations:** ^1^ Department of Small Animal Clinical Sciences Virginia‐Maryland College of Veterinary Medicine Blacksburg Virginia USA; ^2^ VCA Arboretum View Animal Hospital Downers Grove Illinois USA

**Keywords:** bile duct obstruction/rupture, hepatology, hyperbilirubinemia, pancreas

## Abstract

**Background:**

Pancreatitis is a common cause of extrahepatic bile duct obstruction (EHBDO) in dogs. Information describing the clinical course of dogs with pancreatitis associated bile duct obstruction (PABDO) is limited.

**Objectives:**

To describe the clinical course of PABDO in dogs and determine if presumed markers of disease severity are predictors of survival.

**Animals:**

Forty‐six client‐owned dogs with PABDO.

**Methods:**

A retrospective review of medical records from dogs diagnosed with PABDO was performed. Data, including clinical signs and biochemical changes, were collected 6 times throughout the course of disease. Outcome was defined as either survival (discharge from the hospital) or death.

**Results:**

Thirty‐three (79%) out of 42 dogs with PABDO survived. Thirty‐one (94%) of the 33 dogs that survived received medical management alone. Time from onset of clinical signs to initial documented increase in serum bilirubin concentration, peak bilirubin elevation, and initial decline in serum bilirubin concentration were 7 (median), 8, and 15 days, respectively. The median number of days from onset of clinical signs to outcome date was 13. Clinical signs of fever, vomiting, and anorexia were decreased in frequency from the onset of clinical signs to the time of peak bilirubin. Median bile duct dilatation at the time of ultrasonographic diagnosis of PABDO and peak bilirubin were not different between survivors (7.6 mm, 11.7 mg/dL) and nonsurvivors (6 mm, 10.6 mg/dL, *P* = .12, *P* = .8).

**Conclusions:**

Dogs with PABDO often have a prolonged course of illness and improve clinically despite biochemical evidence of progression of EHBDO.

**Abbreviations**
ALP: alkaline phosphatase
ALT: alanine aminotransferase
BUN: blood urea nitrogen
EHBDO: extrahepatic bile duct obstruction
PABDO: pancreatitis associated bile duct obstruction
WBC: white blood cell count

## 1 INTRODUCTION

Pancreatitis is a common cause of extrahepatic bile duct obstruction (EHBDO) in dogs and cats, accounting for 42% of cases in 1 study.^1^ Bile duct obstruction can result in clinically important complications including derangements of hemostasis,^2^, ^3^ hepatic injury,^4^ alterations in hepatic circulation,^5^ reduced liver reticuloendothelial activity,^6^ gallbladder wall necrosis, and gallbladder rupture.^7^ Various treatments have been described for dogs with pancreatitis associated bile duct obstruction (PABDO), including biliary decompression procedures and supportive medical treatment. Biliary decompression can be achieved with percutaneous ultrasound‐guided cholecystocentesis, endoscopic retrograde biliary stenting, or surgical intervention, such as cholecystoduodenostomy or choledocal stent placement.^8^, ^9^, ^10^ Surgery of the biliary tract carries substantial risk in dogs with pancreatitis, with a case fatality rate of 50% in dogs.^11^ Complications associated with cholecystocentesis are uncommon but can include bile peritonitis and cardiorespiratory arrest.^12^ The authors' clinical impression is that most dogs recover without biliary decompression. Unfortunately, few reports describe the clinical course and outcome of dogs with PABDO.^8^, ^13^ The primary aim of our study was to describe the clinical course of PABDO and determine if presumed markers of disease severity, including degree of bile duct dilatation, serum concentrations of bilirubin, urea (BUN), creatinine, and albumin, serum activities of alanine aminotransferase (ALT) and alkaline phosphatase (ALP), white blood cell count (WBC) and platelet counts are predictors of survival to discharge from the hospital.

## 2 MATERIALS AND METHODS

### 2.1 Case selection

Medical records of dogs that were diagnosed with PABDO at the Virginia‐Maryland College of Veterinary Medicine (VMCVM) were identified by review of medical records for cases presenting from February 1999 to January 2017. Terms used during the medical record search included “obstruction bile duct: biliary passage,” “fibrosis bile duct: biliary passage,” “disease bile duct due to unknown,” “obst bile duct,” and “pancreatitis.” Dogs were diagnosed with pancreatitis based on a point scoring system (Table 1). Dogs with a pancreatitis score ≥ 10 were considered to have pancreatitis. A diagnosis of EHBDO was made if the patient had a plasma bilirubin concentration ≥ 2.0 mg/dL on at least 1 occasion and ultrasonographic evidence of bile duct dilatation (common bile duct diameter greater than 3 mm). Ultrasound images from each case were reviewed by a board‐certified radiologist (M. M. L.). Cases were excluded if another cause of hyperbilirubinemia (ie, hemolysis or primary hepatic disease) or concurrent gallbladder mucocele, biliary neoplasia, or obstructive cholelithiasis was identified.

**TABLE 1:** Pancreatitis scoring system

1 point	Clinical findings: Vomiting Pyrexia Cranial abdominal pain Biochemical abnormalities: Leukocytosis Neutrophilic left shift Increased alanine aminotransferase Increased alkaline phosphatase Increased total bilirubin Abdominal ultrasound abnormalities: Peripancreatic free fluid Pancreatic nodule(s) (heteroechoic pancreas)
2 points	Abdominal ultrasound abnormalities: Pancreatic enlargement Duodenal changes (corrugation, ileus, or poor layer definition)
3 points	Abdominal ultrasound abnormalities: Hyperechoic mesenteric fat Irregular pancreatic margins Hypoechoic pancreas
≥10 points	Clinical diagnosis of pancreatitis

*Note:* A score greater than or equal to 10 is consistent with a diagnosis of pancreatitis.

Abbreviation: PABDO, pancreatitis associated bile duct obstruction.

### 2.2 Data collection

Data were collected at 6 time points during the course of illness (Table 2). The time at which blood samples were collected and ultrasound was performed was not standardized because of the retrospective nature of the study. Outcome was defined as either survival (discharge from the Veterinary Teaching Hospital) or death. Clinical signs (vomiting, diarrhea, abdominal pain, anorexia, and fever > 102.5 F) and results of serum chemistry and complete blood cell count were recorded at each time period when available. Complete blood cell counts and serum biochemistry could have been performed at a primary care hospital or the Veterinary Teaching Hospital. Ultrasonographic information collected included common bile duct diameter and whether the following were present: peripancreatic fluid, abnormalities in the duodenum, hyperechoic mesenteric fat surrounding the pancreas, irregular pancreatic margins, hypoechoic appearance of pancreas, pancreatic enlargement, and pancreatic nodules. Other data evaluated included age, breed, sex, major concurrent disease, outcome date, reason for euthanasia or death if applicable, and whether biliary decompression (ie, surgical or cholecystocentesis) was performed.

**TABLE 2:** Time points at which clinicopathologic data from dogs with pancreatitis associated bile duct obstruction were collected

0	Owner reported onset of clinical signs
1	Initial presentation to a veterinarian for assessment
2	Initial documented serum bilirubin elevation (≥2 mg/dL)
3	Ultrasonographic diagnosis of PABDO
4	Highest recorded serum bilirubin
5	Initial decline in serum bilirubin by at least 1 mg/dL

Abbreviation: PABDO, pancreatitis associated bile duct obstruction.

### 2.3 Statistical analysis

Normal probability plots were performed to determine whether continuous variables were skewed. If skewed, continuous variables were summarized as medians (range), while contingency tables were generated for the categorical variables. Nonsurvivors were compared to survivors using the Wilcoxon rank sum test for bile duct dilatation at time point 3 and for WBC, platelet count, BUN, creatinine, albumin, ALT, ALP, total bilirubin, and cholesterol separately at time point 1 and at time point 4. Blood counts (WBC, neutrophils, and band neutrophils), biochemical variables (total bilirubin, ALP, ALT, and cholesterol, BUN, creatinine, albumin), and days since onset of clinical signs were compared between time points (within dog) using Friedman repeated measures ANOVA by ranks test followed by Bonferroni's procedure for multiple comparisons. Presence of clinical signs (fever, anorexia, vomiting, diarrhea, and abdominal pain), 1 at a time, was compared between time points using Mantel‐Haenszel chi‐square test followed by Bonferroni's procedure for multiple comparisons. Statistical significance was set to *P* ≤ .05. All analyses were performed using SAS version 9.4 (Cary, North Carolina).

## 3 RESULTS

Forty‐six dogs met inclusion criteria for the study, including 24 spayed females and 22 neutered males. Eighteen breeds were included in our study; mixed breed (n = 15), miniature schnauzer (n = 4), Yorkshire terrier (n = 3), Chihuahua (n = 2), dachshund (n = 2), golden retriever (n = 2), Labrador retriever (n = 2), miniature poodle (n = 2), miniature pinscher (n = 2), pug (n = 2), Siberian husky (n = 2), Shetland sheepdog (n = 2), and a single dog of the following: Bouvier des Flandres, Jack Russell terrier, Chinese crested, Lhasa apso, Norwegian elkhound, and shih tzu. The median age was 9 years (range 2‐15). The average pancreatitis score was 14.5 with a range of 10 to 21 and all dogs had ultrasonographic evidence of pancreatitis that contributed points to their pancreatitis score. Major concurrent disease included diabetes mellitus (n = 5), cardiac arrhythmia (n = 5), renal failure (n = 3), idiopathic epilepsy (n = 3), thyroid carcinoma (n = 1), and portal vein thrombus (n = 1).

Thirty‐three (79%) out of 42 dogs survived whereas 9 (21%; 95% CI = 10%‐34%) died. Dogs that did not have time 5 data available and were lost to follow‐up (n = 4) were excluded from survival analysis. Three dogs that did not have time 5 data died shortly after discharge (n = 3) and were considered nonsurvivors in the survival analysis. Five dogs did not have time 5 data but lived years after discharge and were included as survivors. All dogs that died were euthanized. Contributing factors for euthanasia included persistent clinical signs associated with pancreatitis (n = 3), acute renal failure (n = 2), cardiac arrhythmias (n = 2), pancreatic atrophy (n = 1), and bile peritonitis (n = 1). Biliary decompression was performed in 4 of 46 dogs (9%). One dog that received a cholecystoduodenostomy developed acute renal failure after undergoing cardiopulmonary arrest postoperatively and was euthanized. Percutaneous ultrasound guided cholecystocentesis was performed on the remaining 3 patients that received biliary decompression, 1 of which developed bile peritonitis and was euthanized. Of the 33 dogs that survived, 31 (94%) were not treated with a biliary decompression procedure. One dog presented twice for PABDO 1 year apart and survived to discharge each time without biliary decompression. Time 5 data were not available for the second time the patient presented. Because of the retrospective nature of the study, treatment was not standardized and could include crystalloid treatment, antibiotic treatment, analgesics, antiemetics, gastroprotectants, and fresh frozen plasma. Survival analysis in relation to treatment was not assessed.

The median number of days from time 0 to times 1, 2, 3, 4, and 5 was 2 days (range, 0‐16), 7 days (range, 0‐24), 8 days (range, 1‐22), 9 days (range, 0‐27), and 15 days (range, 5‐31), respectively. Data for time 5 were available in only 29 cases. All but 1 of the dogs that died in hospital were euthanized and did not survive until time point 5. Twelve dogs were discharged before initial decline in total serum bilirubin concentration. Five of these dogs lived years after discharge, 3 were euthanized shortly after discharge, and 4 were lost to follow‐up. The median number of days from onset of clinical signs to outcome date was 13 (1‐41).

Clinical signs of fever, vomiting, and anorexia were significantly less prevalent by time 4 (Table 3). Abdominal pain was less prevalent between time 3 and 5 (Table 3). Median ALP activity increased significantly up to time 4 and declined at time 5 (Table 4). Median ALT activity significantly increased from time 1 to 2 and decreased from time 3 to 5. White blood cells did not significantly change over time. Median BUN was decreased at time 2, 3, 4, and 5 compared to time 1. Median creatinine decreased from time 1 to 4 and median albumin was decreased at time 3, 4, and 5 compared to time 1. When the clinicopathologic data for dogs that had time 5 data available (n = 29) were analyzed separately, no difference in statistical significance was noted when compared with all 46 dogs. Median WBC and BUN concentrations were higher in nonsurvivors and albumin was lower in nonsurvivors compared with survivors at time 1 (Table 5). At time 4, ALT was significantly higher in survivors compared to nonsurvivors and albumin was significantly lower in nonsurvivors (Table 5).

**TABLE 3:** Prevalence of clinical signs in 46 dogs with pancreatitis associated bile duct obstruction

Time point:	1	2	3	4	5	*P* value
Fever	13/42 (31%)^a,b^	8/39 (21%)	6/39 (15%)	2/43 (5%)^a^	1/28 (4%)^b^	<.001
Anorexia	41/45 (91%)^a,b^	31/38 (82%)^c^	24/34 (71%)	23/40 (58%)^a^	10/28 (36%)^b,c^	<.001
Vomiting	41/45 (91%)^a,b,c^	29/42 (69%)^d^	16/39 (41%)^a,e^	15/46 (33%)^b^	4/27 (15%)^c,d,e^	<.001
Diarrhea	13/45 (29%)	8/41 (20%)	9/39 (23%)	6/45 (13%)	5/28 (18%)	.18
Abdominal pain	20/41 (49%)	16/41 (39%)	20/38 (53%)^a^	19/44 (43%)	4/26 (15%)^a^	.007

*Note:* Superscript letters that are the same within each row indicate significant differences between time points based on Bonferroni's procedure for multiple comparisons. The level of significance is set at *P* ≤ .05. See Table 2 for description of time points.

**TABLE 4:** Selected clinicopathologic variables at each time in dogs with pancreatitis associated bile duct obstruction

Variable	Time	n	Median (range)	*P* value
White blood cell count (×10^3^)	1	42	16.2 (4‐53.4)	.09
	2	37	16.1 (4‐38.4)	
	3	31	17.8 (8.3‐45.8)	
	4	30	20.5 (7.4‐45.8)	
	5	12	19.4 (11.4‐41.5)	
Neutrophils (×10^3^)	1	42	12.3 (0.9‐44.3)	.03
	2	37	13.1 (0.9‐35.5)	
	3	31	16 (5.2‐40.3)	
	4	30	17.3 (5.2‐40.3)	
	5	12	16.7 (8.3‐36.1)	
Band neutrophils	1	38	0 (0‐8822)	.17
	2	34	0 (0‐8822)	
	3	30	0 (0‐1832)	
	4	29	0 (0‐1404)	
	5	11	0 (0‐758)	
Platelets (×10^3^)	1	38	314 (12‐829)	.55
	2	33	313 (12‐569)	
	3	28	322.5 (0‐655)	
	4	29	303 (113‐655)	
	5	12	354 (224‐633)	
Bilirubin (mg/dL)	1	44	3.1 (0.1‐27.9) ^a,b,c^	<.001
	2	46	7.0 (2.2‐27.9) ^a,d,e^	
	3	36	8.9 (1.6‐27.9) ^b,f,g^	
	4	46	11.3 (2.7‐33.2) ^c,d,f^	
	5	29	2.4 (0.6‐13.7) ^e,g^	
Alkaline phosphatase (U/L)	1	40	1502 (80‐5456)^a,b,c,d^	<.001
	2	38	2000 (486‐10 966)^a^	
	3	35	4155 (521‐16 056)^b^	
	4	42	4288 (304‐14 973)^c^	
	5	27	3202 (580‐16 655)^d^	
Alanine aminotransferase (U/L)	1	38	128.5 (28‐4000)^a^	<.001
	2	40	926.5 (28‐4000)^a^	
	3	35	1000 (139‐2702)^b,c^	
	4	43	934 (165‐2702)^b^	
	5	26	581 (78‐2351)^c^	
Cholesterol (mg/dL)	1	28	411.5 (96‐556)	.002
	2	33	492 (122‐1178)	
	3	33	584 (209‐1001)	
	4	40	588 (207‐1001)	
	5	26	459 (136‐992)	
Blood urea nitrogen (mg/dL)	1	44	14 (5‐196)^a,b,c,d^	<.001
	2	45	9.2 (4‐196)^a^	
	3	36	9.0 (4‐123)^b^	
	4	44	8.0 (4‐123)^c^	
	5	25	9.0 (4‐19)^d^	
Creatinine (mg/dL)	1	42	0.9 (0.5‐7.7)^a^	.01
	2	41	0.9 (0.4‐7.7)	
	3	36	0.7 (0.3‐5.3)	
	4	43	0.8 (0.3–5.3)^a^	
	5	25	0.7 (0.4‐2.2)	
Albumin (mg/dL)	1	40	3.1 (1.5‐27)^a,b,c^	<.001
	2	44	2.7 (1.5‐4.1)	
	3	35	2.8 (1.1‐3.6)^a^	
	4	44	2.7 (1.1–3.6)^b^	
	5	26	2.8 (1.7‐3.3)^c^	

*Note:* Superscript letters that are the same within each column indicate significant differences between time points based on Bonferroni's procedure for multiple comparisons. The level of significance is set at *P* ≤ .05. See Table 2 for description of time points.

**TABLE 5:** Selected clinicopathologic variables compared between survivors and nonsurvivors in dogs with pancreatitis associated bile duct obstruction

Time	Variable	Group	n	Median (range)	*P* value
1	White blood cell count (×10^3^)	Died	8	26.2 (13.4‐36.1)	.03
		Survived	31	15 (4‐53.4)	
	Platelets	Died	8	382.5 (133‐575)	.43
		Survived	27	306 (12‐829)	
	Alanine aminotransferase (U/L)	Died	8	249 (87‐1057)	.72
		Survived	28	115 (28‐4000)	
	Alkaline phosphatase (U/L)	Died	8	581 (183‐2869)	.45
		Survived	30	1567 (80‐5456)	
	Bilirubin (mg/dL)	Died	9	1.2 (0.3‐19.8)	.78
		Survived	32	3.3 (0.1‐27.9)	
	Cholesterol (mg/dL)	Died	6	417 (235‐556)	.7
		Survived	20	407 (96‐521)	
	Blood urea nitrogen (mg/dL)	Died Survived	9 32	21 (8‐196) 13 (5‐87)	.04
	Creatinine (mg/dL)	Died Survived	9 30	1.2 (0.5‐7.7) 0.9 (0.5‐5.8)	.08
	Albumin (mg/dL)	Died Survived	9 29	2.7 (1.5‐3.4) 3.3 (2‐4.5)	.02
4	White blood cell count (×10^3^)	Died	7	21.2 (15‐45.8)	.5
		Survived	20	18.8 (7.4‐35.1)	
	Platelets	Died Survived	7 19	300 (113‐600) 313 (117‐655)	.55
	Alanine aminotransferase (U/L)	Died	9	787 (165‐1234)	.05
		Survived	30	1062.5 (200‐2702)	
	Alkaline phosphatase (U/L)	Died	9	4450 (610‐11 382)	.71
		Survived	29	4320 (304–14 973)	
	Bilirubin (mg/dL)	Died	9	10.6 (4.7‐23.3)	.8
		Survived	33	11.7 (2.7‐33.2)	
	Cholesterol (mg/dL)	Died	9	541 (265‐881)	.47
		Survived	29	589 (207‐1001)	
	Blood urea nitrogen (mg/dL)	Died Survived	9 31	14 (4‐123) 8 (4‐75)	.21
	Creatinine (mg/dL)	Died Survived	9 30	0.7 (0.6‐5.3) 0.8 (0.3‐2.6)	.95
	Albumin (mg/dL)	Died Survived	9 31	2.4 (1.1‐2.7) 2.9 (1.6‐3.6)	.009

*Note:* The level of significance is set at *P* ≤ .05.

The median common bile duct diameter was 6.5 mm (4‐13), and was not significantly different (*P* = .12) between survivors (7.6 mm, 4‐13) and nonsurvivors (6 mm, 4‐10). The presence of ultrasonographic abnormalities of the pancreas and duodenum are summarized in Table 4. A single ultrasound examination was performed in most dogs so serial measurements were not included in our study (Table 6).

**TABLE 6:** Ultrasonographic findings in 46 dogs with pancreatitis associated bile duct obstruction

Ultrasonographic finding	n (%)
Peripancreatic fluid	11 (24)
Duodenal changes	18 (39)
Hyperechoic mesenteric fat	39 (87)
Irregular pancreatic margins	31 (67)
Hypoechoic pancreas	42 (91)
Pancreatic nodules	14 (30)
Increased pancreatic size	26 (56)

## 4 DISCUSSION

Although pancreatitis is a common cause of EHBDO in dogs,^1^ there is minimal information available to date describing the clinical course of this illness. Findings of our study provide further evidence that pancreatitis can cause sustained inflammation adjacent to the common bile duct that leads to a functional or physical obstruction over time. In our population of dogs, the highest recorded bilirubin, which likely represents the peak of bile duct obstruction, occurred a median of 9 days after the onset of clinical signs. A prolonged period of recovery should be expected in dogs with PABDO since initial decline in bilirubin did not occur until a median of 15 days from onset of signs. A previous description of PABDO described an onset of icterus similar to our study. However, dogs in the previous study had mass lesions in their pancreas and evidence of chronic active fibrosing pancreatitis on histopathology.^8^ Interestingly, many of the clinical signs resulting from pancreatitis, including anorexia, vomiting, and fever, had decreased by the time peak bilirubin occurred in our population. We suspect the initial inflammatory response associated with acute pancreatitis, which usually includes acinar cell damage and necrosis, leukocyte migration, and cytokine release, is resolving as the EHBDO develops.^14^ It seems the clinical signs observed in dogs with PABDO *might* primarily be a result of pancreatitis rather than bile duct obstruction. Additionally, hyperbilirubinemia *might* persist despite resolution of biliary obstruction since conjugated bilirubin binds covalently to albumin and delays the clearance of bilirubin.^15^

Overall survival for dogs with PABDO was 79% in our population, with only 7 dogs treated with medical management alone not surviving. Two of the 4 dogs that had percutaneous or surgical decompression of the gallbladder died. The case fatality rate of dogs undergoing surgery in previous reports of EHBDO from various causes ranges from 17% to 58%.^1^, ^8^, ^11^, ^13^ A case fatality rate of 50% has been reported in dogs with EHBDO secondary to pancreatitis undergoing surgical biliary decompression and choledochal stent placement.^9^, ^11^ Results of our study show that most dogs will recover with medical management alone. Percutaneous ultrasound guided cholecystocentesis is an alternative to surgical biliary decompression but carries its own risk. Although uncommon, complications occur in 2.7% of dogs undergoing cholecystocentesis including bile peritonitis, SIRS, and cardiorespiratory arrest.^12^ One of the 3 dogs in our population that received cholecystocentesis developed bile peritonitis and was ultimately euthanized. Most dogs with PABDO described herein experienced a prolonged course of illness but ultimately recovered without biliary decompression.

It is sometimes difficult to differentiate whether dogs have acute or chronic pancreatitis based on clinical signs alone. Six dogs with presumed acute PABDO based on clinical signs had evidence of chronic pancreatitis and fibrosis on pancreatic histopathology. Each of these dogs had a firm mass involving the body of the pancreas that was a result of chronic pancreatitis. Interestingly, these dogs developed icterus 2 to 14 days after onset of signs of acute pancreatitis, which is similar to the results we obtained.^8^ Dogs in our study had signs most consistent with acute PABDO based on duration of signs. Thirty percent of the dogs had pancreatic nodules on ultrasound though, which could be suggestive of a chronic component since mass lesions have been reported in dogs with chronic pancreatitis.^16^ We cannot definitively determine whether our population of dogs was experiencing acute or chronic pancreatitis since histopathology was not obtained.

Markers of bile duct obstruction, including peak total bilirubin and bile duct dilatation, were not predictors of survival in our population of dogs. Based on this observation and a favorable survival rate with medical management alone, we conclude that factors such as degree of common bile duct dilatation and hyperbilirubinemia should not dictate whether surgical intervention or cholecystocentesis is performed. In humans, surgical biliary decompression is usually reserved for people with bile duct stricture secondary to chronic pancreatitis rather than acute pancreatitis. Surgical biliary drainage in humans is recommended when jaundice has been present for greater than 1 month.^17^ In our population of dogs, the onset of clinical signs to outcome date was longer than a month in only 2 dogs.

Our study has several limitations. Because of the retrospective nature, accurate interpretation of clinical signs at different time points was dependent upon accurate documentation. The total bilirubin was also not measured at standardized time points, so it is possible that the actual peak bilirubin was missed in some cases. Seven of the dogs in our study had their peak bilirubin recorded at the onset of their clinical signs, making time point 1 and 4 appear similar for purposes of the study. The initial decline of total bilirubin was also not available for every case. The exact timeline of PABDO was not unequivocally established in our study as the times of evaluation varied, the time point at which the abdominal ultrasound was performed was also not standardized, and serial ultrasounds were not performed because of the retrospective nature of this study. Intrahepatic bile duct dilatation, to assess for chronicity of biliary obstruction, was also not measured. Supportive medical treatment was not standardized and could include IV fluid treatment, antibiotic treatment, analgesics, antiemetics, gastroprotectants, and fresh frozen plasma. Whether particular therapies, such as fresh frozen plasma, improved outcome in dogs that did not receive biliary decompression is unknown. All dogs that died were euthanized, which could have affected interpretation of survival.

Since pancreatitis can be challenging to definitively diagnose, we attempted to minimize ambiguity by creating a pancreatitis scoring system. Histopathology is usually required to obtain a definitive diagnosis. Unfortunately, histopathology is impractical in a clinical setting and has its own limitations because pancreatitis often has a patchy distribution and the clinical relevance of inflammatory lesions is often difficult to determine.^18^ Although various assays for pancreatic lipase are often highly sensitive, they have variable specificity.^19^ The scoring system was designed to assign points for clinical signs, clinicopathologic changes, and ultrasonographic abnormalities that are typically associated with pancreatitis in dogs.^20^, ^21^ In addition to their variable specificity, assays for pancreatic lipase were not included in our scoring system because they were not consistently assessed in our population of dogs. Scoring systems are frequently used in humans with acute pancreatitis for diagnosis and assessing disease severity but there is not currently a universally accepted pancreatitis scoring system in dogs.^22^ For example, some authors might use a combination of clinical signs, serum pancreatic lipase concentration, and abdominal ultrasound to make the diagnosis of acute pancreatitis,^23^ while others depend heavily upon expert opinion.^19^

## CONFLICT OF INTEREST DECLARATION

Authors declare no conflict of interest.

## OFF‐LABEL ANTIMICROBIAL DECLARATION

Authors declare no off‐label use of antimicrobials.

## INSTITUTIONAL ANIMAL CARE AND USE COMMITTEE (IACUC) OR OTHER APPROVAL DECLARATION

Authors declare no IACUC or other approval was needed.

## HUMAN ETHICS APPROVAL DECLARATION

Authors declare human ethics approval was not needed for this study.
